# Meta‐analysis of the effects of sodium glucose cotransporter 2 inhibitors in non‐alcoholic fatty liver disease patients with type 2 diabetes

**DOI:** 10.1002/jgh3.12473

**Published:** 2020-12-07

**Authors:** Binayak Sinha, Debasis Datta, Samit Ghosal

**Affiliations:** ^1^ Department of Endocrinology, AMRI Hospitals Kolkata India; ^2^ Department of Hepatology, Fortis Hospital Kolkata India; ^3^ Department of Endocrinology, Nightingale Hospital Kolkata India

**Keywords:** alanine aminotransferase, aspartate aminotransferase, liver fat, non‐alcoholic fatty liver disease, sodium glucose cotransporter 2 inhibitors, type 2 diabetes

## Abstract

**Background and Aim:**

Sodium glucose cotransporter 2 inhibitors (SGLT‐2i), by way of their unique mode of action, present an attractive strategy for the treatment of type 2 diabetes and non‐alcoholic fatty liver disease (NAFLD), which often coexist and may lead to severe complications. However, the evidence for treatment with SGLT‐2i is limited to small heterogeneous studies. Therefore, this meta‐analysis was conducted to deduce the effects of SGLT‐2i in NAFLD with type 2 diabetes (T2D).

**Methods:**

A web‐based search identified nine randomized controlled trials from the Cochrane Library, Embase, and PubMed for this meta‐analysis. The Comprehensive Meta‐Analysis Software version 3 was used to calculate the effect size.

**Result:**

The outcomes of interest were analyzed from a pooled population of 11 369 patients—7281 on SGLT‐2i and 4088 in the control arm. SGLT‐2i therapy produced a statistically significant improvement in alanine aminotransferase [standardised mean difference (SDM), −0.21, 95% confidence interval (CI), −0.32 to −0.10, *P* < 0.01], aspartate aminotransferase (Standardised mean difference (SDM), −0.15, 95% CI, −0.24 to −0.07, *P* < 0.01), and liver fat as measured by proton density fat fraction (SDM, −0.98, 95% CI, −1.53 to −0.44, *P* < 0.01) in comparison to standard of care or placebo. In addition, there was a significant reduction in glycosylated hemoglobin (SDM, −0.37, 95% CI, −0.60 to −0.14, *P* < 0.01) and weight (SDM, −0.58, 95% CI, −0.93 to −0.23, *P* < 0.01) in the SGLT‐2i arm.

**Conclusion:**

This meta‐analysis provides a convincing signal that SGLT‐2i have a salutary effect on NAFLD in type 2 diabetes (T2D), probably driven by an improvement of glycemia and body weight, which in turn attenuates hepatic inflammation and hepatic fat accumulation.

## Graphical abstract

Sodium glucose cotransporter 2 inhibitors possess the capability to improve glycemic status along with weight reduction in patients with type 2 diabetes with non‐alcoholic fatty liver disease. As a result, there is a possibility that it may effect an improvement in preexisting hepatic inflammatory states. This meta‐analysis assessed such a possibility and found a positive influence.

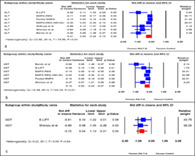

## Introduction

Type 2 diabetes (T2D) and non‐alcoholic fatty liver disease (NAFLD) are two very common conditions that frequently coexist.^1^, ^2^ These conditions may worsen the outcomes of both T2D (macro‐ and microvascular complications)^3^, ^4^ and NAFLD (cirrhosis and hepatocellular carcinoma) synergistically.^5^, ^6^, ^7^ Therefore, treatment should be initiated early and aggressively to prevent these serious complications.

The mainstay of the management of NAFLD with T2D is achieving good metabolic control and weight loss.^8^ Although some degree of success is achieved through lifestyle management, in many patients, lifestyle management is not enough to stem the severity of these conditions.^9^

Sodium glucose cotransporter 2 inhibitors (SGLT‐2i) are a new class of oral antidiabetics that act by decreasing renal glucose reabsorption. The effect of increased renal glucose excretion serves the dual purpose of glycemic control and calorie loss, translating into a weight loss of approximately 2 kg. Interestingly, this weight loss is derived mainly from the loss of fat mass rather than from osmotic diuresis.^10^, ^11^ In addition, animal models of NAFLD with SGLT‐2i have demonstrated a protective effect on steatosis, inflammation, and fibrosis.^12^, ^13^ Therefore, this represents an attractive strategy for the treatment of NAFLD with T2D. Multiple studies addressing the use of SGLT‐2i in this dual disease are unfortunately limited by their small sample sizes, heterogeneous inclusion criteria, and primary outcomes, as well as duration of follow‐up, thus making it almost impossible to draw robust conclusions that are applicable across the entire spectrum of NAFLD and diabetes.^14^, ^15^, ^16^, ^17^, ^18^, ^19^, ^20^, ^21^, ^22^, ^23^ A recent meta‐analysis of SGLT‐2i inhibitors showed a significant decrease in alanine aminotransferase (ALT) and liver fat, accompanied by weight loss, but was limited by the extremely small sample size.^24^

In the absence of robust data, this meta‐analysis was conducted to decipher in detail and provide clarity on the effects of SGLT‐2i in NAFLD with T2D.

## Methods

### *Search and study selection process*

**Figure 1:**
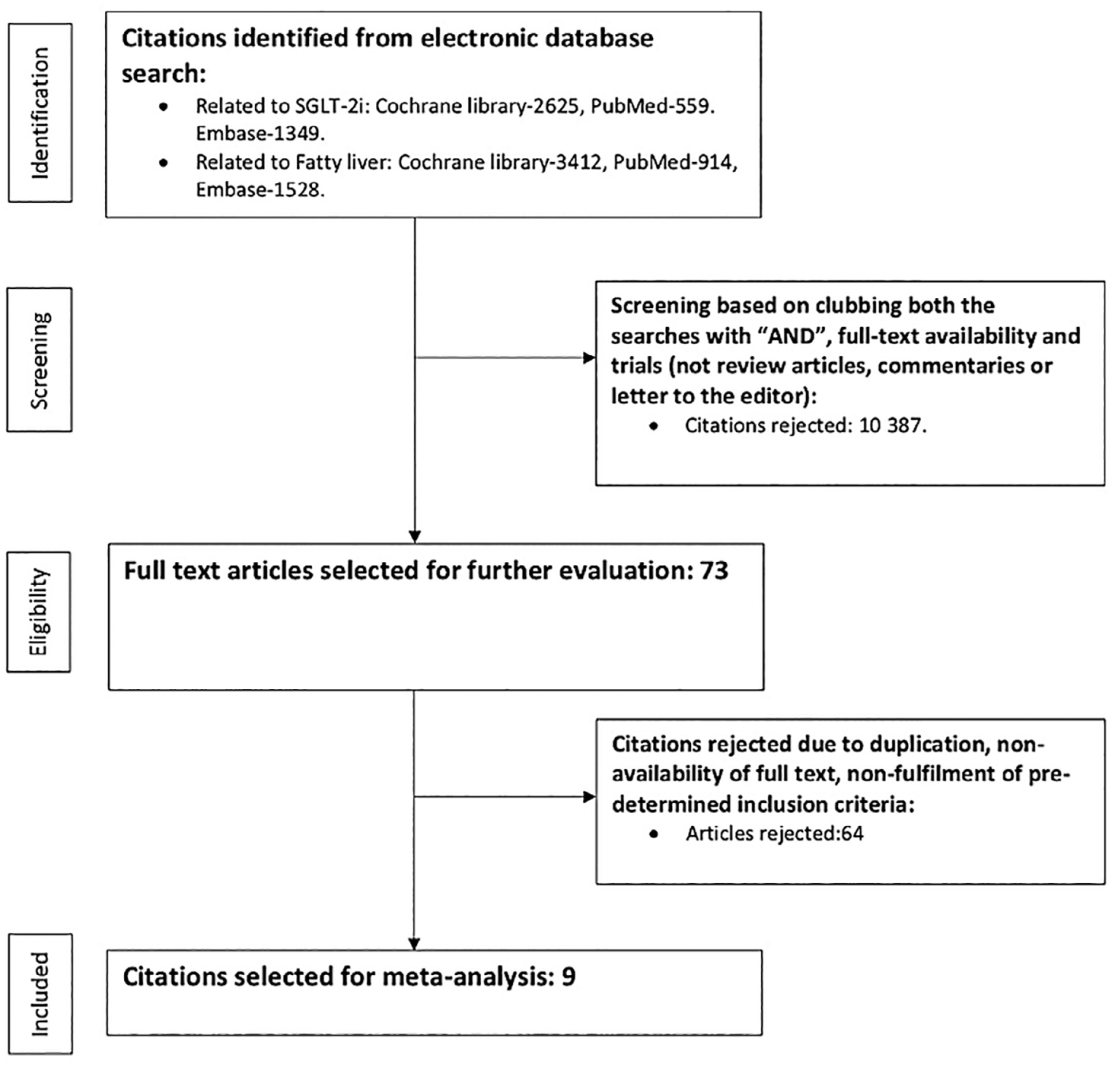
Study selection process. SGLT‐2i, sodium glucose cotransporter 2 inhibitors.

A thorough web‐based search (Cochrane library, PubMed, and Embase) was conducted by the authors (Debasis Datta, Binayak Sinha, and Samit Ghosal) using specific keywords. Preliminary search keywords included “Sodium Glucose Transporter 2 Inhibitors” (MeSH) OR SGLT 2 Inhibitors OR SGLT‐2 Inhibitors OR Dapagliflozin OR Empagliflozin OR Ipragliflozin OR Ertugliflozin OR Canagliflozin OR Luseogliflozin as far as SGLT‐2i‐related search was concerned and “non‐alcoholic Fatty Liver Disease” (MeSH) OR NAFLD OR “Fatty Liver” (MeSH), OR “nonalcoholic steatohepatitis” (MeSH) OR NASH for search related to hepatic dysfunction. After the initial broad identification of citations, screening was conducted by Samit Ghosal and Binayak Sinha by studying the SGLT‐2i and hepatic dysfunction citations with the Boolean AND, along with the use of full‐text availability and clinical trials (not review articles, commentaries, letters to the editor, etc.) as additional filters. The eligible citations were screened for a second time by Binayak Sinha and Samit Ghosal by removing duplications and using an inclusion criterion based on consensus (Fig. 1). The inclusion criteria for the final step of the study selection process included:

- Randomized controlled trials.
- Age limit: 18–75 years, with type 2 diabetes mellitus and documented NAFLD.
- Inclusion of a control arm not documented to make any impact on hepatic outcomes.
- A minimum of 12 weeks of follow‐up.
- Reporting of at least two hepatic outcome measures, one inflammatory and another structural in nature.
- Reporting of metabolic outcomes: glycosylated hemoglobin (HbA1C), serum triglycerides (TG), body mass index (BMI), and body weight.
- A clear documentation of exclusion of all non‐NAFLD‐related hepatic dysfunctions

### *Extraction of data including assessment of quality*

Having identified the nine citations to be taken up for analysis, Binayak Sinha and Samit Ghosal extracted the data required for both primary (ALT and aspartate aminotransferase [AST], γ‐glutamyl transferase [GGT], proton density fat fraction [PDFF], visceral fat mass [VFM], liver biopsy parameters) and secondary (HbA1C, weight, BMI, and TG) analysis. The extracted data were entered into an Excel sheet. The entered data were cross‐checked by another author (Debasis Datta) for errors. The quality of the selected citations was assessed using the Cochrane risk‐of‐bias algorithm, which included random sequence generation, allocation concealment, blinding of participants and personnel, blinding of outcome data, incomplete outcome data, selective reporting, and other biases (Figure S1). All the selected citations were evaluated along with their supplementary data and scored individually by Binayak Sinha and Samit Ghosal. Any dispute was reassessed by Debasis Datta, and a final decision was taken by consensus.

### *Patient approval and clearance from the ethical committee*

This study being a systematic review and meta‐analysis, there was no direct handling of patients. In addition, effect size estimates that were already published in open web‐based domains were used to conduct the meta‐analysis. As a result, there was no requirement for patient or ethical committee consent.

### *Statistical analysis*

Standardized mean difference (SMD) was used as the preferred parameter of interest in because of the different reporting patterns in the included citations. Some of the citations reported differences in raw mean without any statistical significance, while others reported the events in both arms only. As four different analytical techniques (independent groups [difference, *P*], raw difference [independent groups, CI], independent groups [standard difference], and independent groups [sample size, *P*]) had to be used to derive the effect size, SMD was used to maintain the uniformity of reporting. In addition to the effect size, hypothesis testing was performed and reported in the form of 95% confidence interval (95% CI) and *P*‐value. A summary of results was reported in the form of a forest plot, which also included the variance and weightage of the individual studies. The analysis was conducted using the Comprehensive Meta‐Analysis (CMA) Software version 3 (Biostat Inc., Englewood, NJ, USA). Heterogeneity was assessed using the Cochrane Q and Higgin's I^2^ test, and publication bias was assessed using funnel plots. Heterogeneity was defined as low (<45%), moderate (45–75%), and high (>75%) based on the I^2^ statistic. All effect sizes were analyzed using the random‐effects model.

A sensitivity and subgroup analysis was planned if significant heterogeneity related to the pooled effect size was encountered. Because of the small number of studies included in this meta‐analysis, sensitivity analysis using the technique of sequential exclusion to identify the study responsible for high heterogeneity was performed. Having done so, a repeat analysis was performed on the same outcome parameter (excluding the culprit citation) to assess replicability of the original finding with minimal or no heterogeneity.

A subgroup analysis was performed using the raw mean difference as the outcome of choice instead of the SMD. Although this resulted in the exclusion of a few studies, the essence of the analysis was not altered.

### *Role of funding*

There was no funding received for preparing this manuscript.

This is a meta‐analysis based on published articles and thus did not qualify for ethics approval.

## Results

### *Baseline characteristics of the studies*

The meta‐analysis was conducted on a pooled patient population of 11 369 from nine citations, divided into 7281 individuals on SGLT‐2i inhibitors and 4088 on standard of care without SGLT‐2i inhibitors or an active control. As part of the inclusion criteria, we excluded studies including antihyperglycemic agents capable of influencing hepatic parameters. For example, an randomised controlled trial (RCT) by Ito *et al*. comparing ipragliflozin *versus* pioglitazone was excluded because of the latter's capability of having a positive impact on NAFLD. Among the nine citations included in the meta‐analysis, EMPA‐REG 2H2‐SU and Shibuya *et al*. had an active control arm in the form of glimepiride and metformin, respectively. The remaining studies had standard‐of‐care antihyperglycemic agents in their control arm without SGLT‐2i, expect for Kahl *et al*. and Eriksson *et al*. where there was a well‐defined placebo arm. The duration of the studies ranged between 12 and 28 weeks. The EMPA‐REG outcomes trial was followed up for 156 weeks. However, to maintain parity as far as the follow‐up periods among all the included citations were concerned, we used the 28‐week follow‐up result. The baseline characteristics of the citations included in the analysis are summarized in Table 1.

**Table 1:** Baseline characteristics of the studies included in the meta‐analysis

Studies (year)	Mean age (years)‐SGLT‐2i group	Gender (male/female)‐SGLT‐2i group	Total patients (SGLT‐2i/control)	Control arm	SGLT‐2i arm	Follow‐up duration (weeks)
*E*‐LIFT‐2018^15^	50.7	16/9	25/25	Standard of care without SGLT‐2i	Empagliflozin 10 mg	20
EMPA‐REG‐2015^16^	63	3336/1351	4687/2333	Standard of care without SGLT‐2i	Empagliflozin 10 mg& 25 mg	28
EMPA‐REG 2H2‐SU 2014^18^	58	420/345	765/780	Glimepiride	Empagliflozin 25 mg	28
Pooled EMPA‐2013 to 2014^3^, ^25^, ^26^, ^27^	55.5–57.4	1024/628	1652/825	Standard of care without SGLT‐2i	Empagliflozin 10 mg& 25 mg	24
Kahl *et al*.‐2020^19^	62.7	29/13	42/42	Placebo	Empagliflozin 25 mg	24
Eriksson *et al*.‐2018^20^	65	16/5	21/21	Placebo	Dapagliflozin 10 mg	12
Shibuya *et al*.‐2017^21^	51	10/6	16/16	Metformin 1.5 g	Luseogliflozin 2.5 mg	24
Bando *et al*.‐2017^22^	54.8	26/14	40/22	Standard of care without SGLT‐2i	Ipragliflozin 50 mg	12
Shimizu *et al*.‐2018^23^	56.2	19/14	33/24	Standard of care without SGLT‐2i	Dapagliflozin 5 mg	24

SGLT‐2i, sodium glucose cotransporter 2 inhibitors.

In most situations, a diagnosis of NAFLD is made on the basis of clinical suspicion and surrogates like transaminases or ultrasonography.^1^, ^25^ This was the criterion used for the diagnosis of NAFLD in all the studies in this meta‐analysis, except EMPA REG and EMPA H2H SU. The latter studies were included in this meta‐analysis as they studied patients with advanced T2D who had atherosclerotic cardiovascular disease, the presence of which is almost a sine qua non of NAFLD.^3^, ^26^ The logical inclusion of these studies enriched our meta‐analysis with a higher number of samples, contrasting with the earlier meta‐analyses, which are restricted by a small sample size.

### *Outcome measures: Liver enzymes*

**Figure 2:**
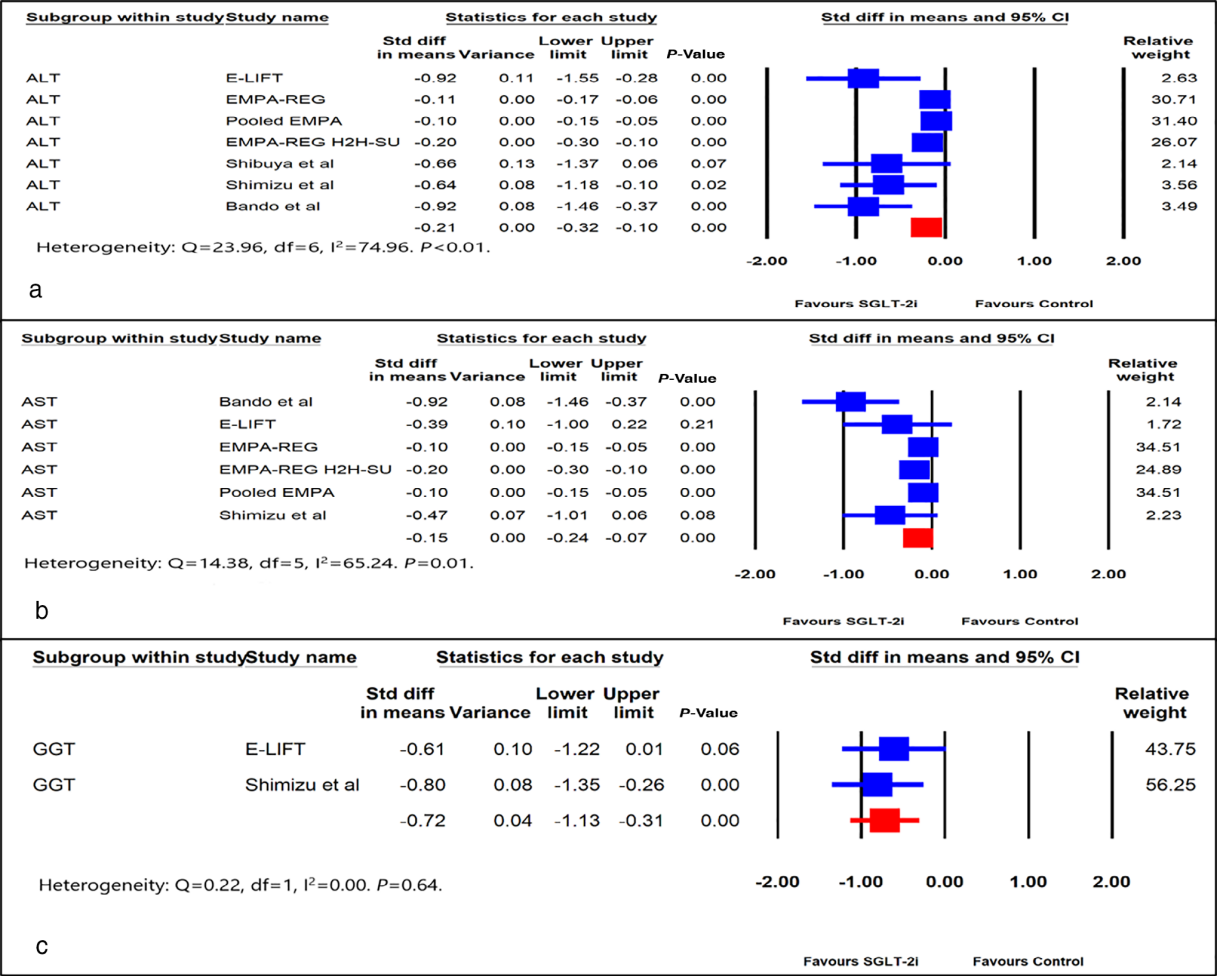
Forest plot comparing effect of SGLT‐2i *versus* control on: (a) alanine aminotransferase (ALT), (b) aspartate aminotransferase (AST), (c) gamma‐glutamyl transferase (GGT). CI, confidence interval; Std, standard.

Seven studies reported ALT, six reported AST, and only two reported GGT. The standardized difference in means of change from baseline for ALT (SDM, −0.21, 95% CI, −0.32 to −0.10, *P* < 0.01) (Fig. 2a), AST (SDM, −0.15, SE, 95% CI, −0.24 to −0.07, *P* < 0.001) (Fig. 2b), and GGT (SDM, −0.72, 95% CI, −1.13 to −0.31, *P* < 0.01) (Fig. 2c) were statistically significant.

### *Outcome measures‐: Liver fat and VFM*

**Figure 3:**
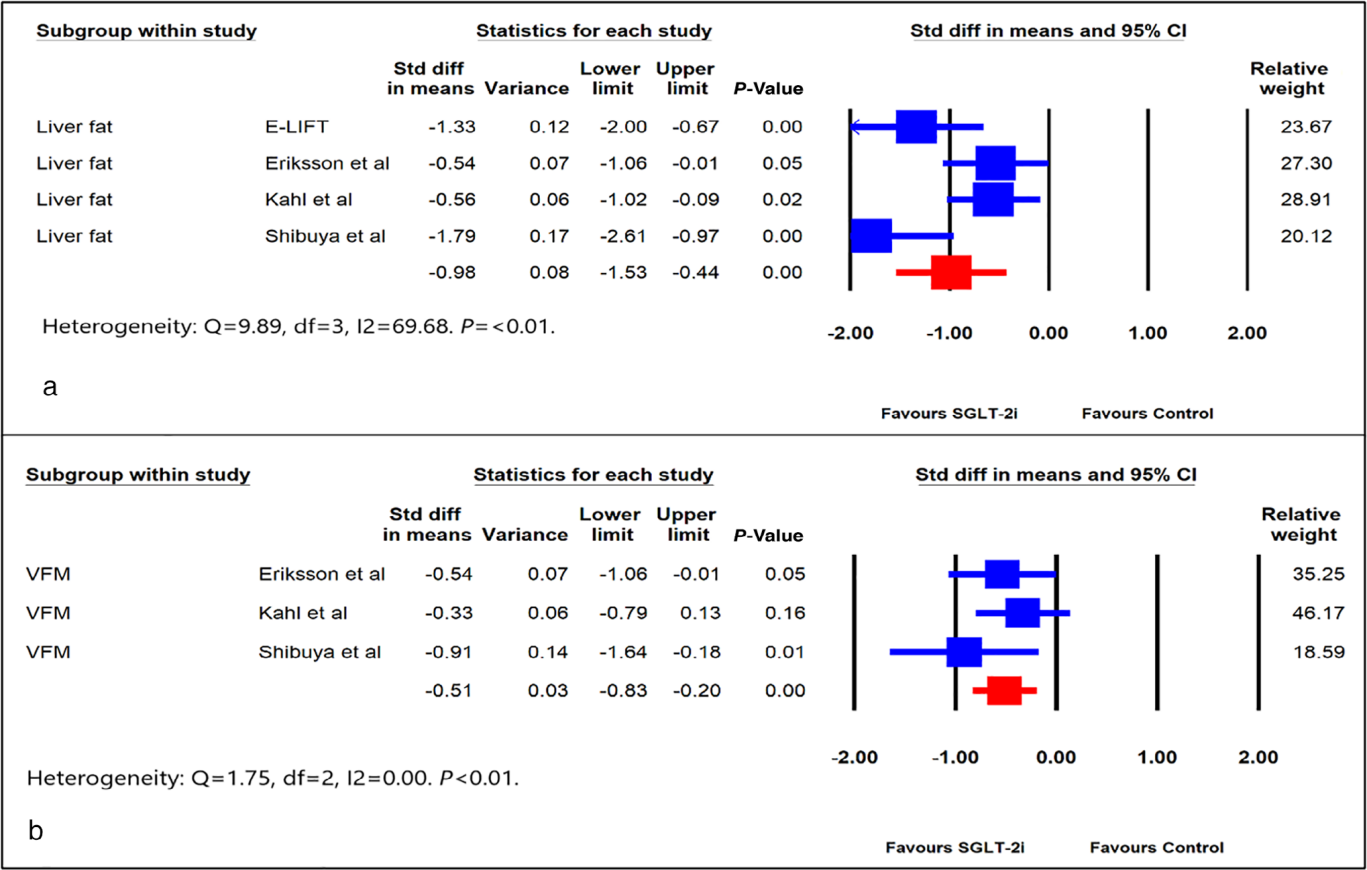
Forest plot comparing effect of sodium glucose cotransporter 2 inhibitors (SGLT‐2i) *versus* control on: (a) Liver fat and (b) visceral fat mass (VFM). CI, confidence interval; Std, standard.

Quantitative assessment of liver fat was assessed by imaging and reported as a PDFF in four of the nine citations, whereas only three citations reported VFM. The standardized differences in the mean change from baseline for liver fat (SDM, −0.98, 95% CI, −1.53 to −0.44, *P* < 0.01) (Fig. 3a) and VFM (SDM, −0.51, SE, 95% CI, −0.83 to −0.20, *P* < 0.01) (Fig. 3b) were statistically significant.

### *Outcome measures: Weight, TG, and HBA1c*

**Figure 4:**
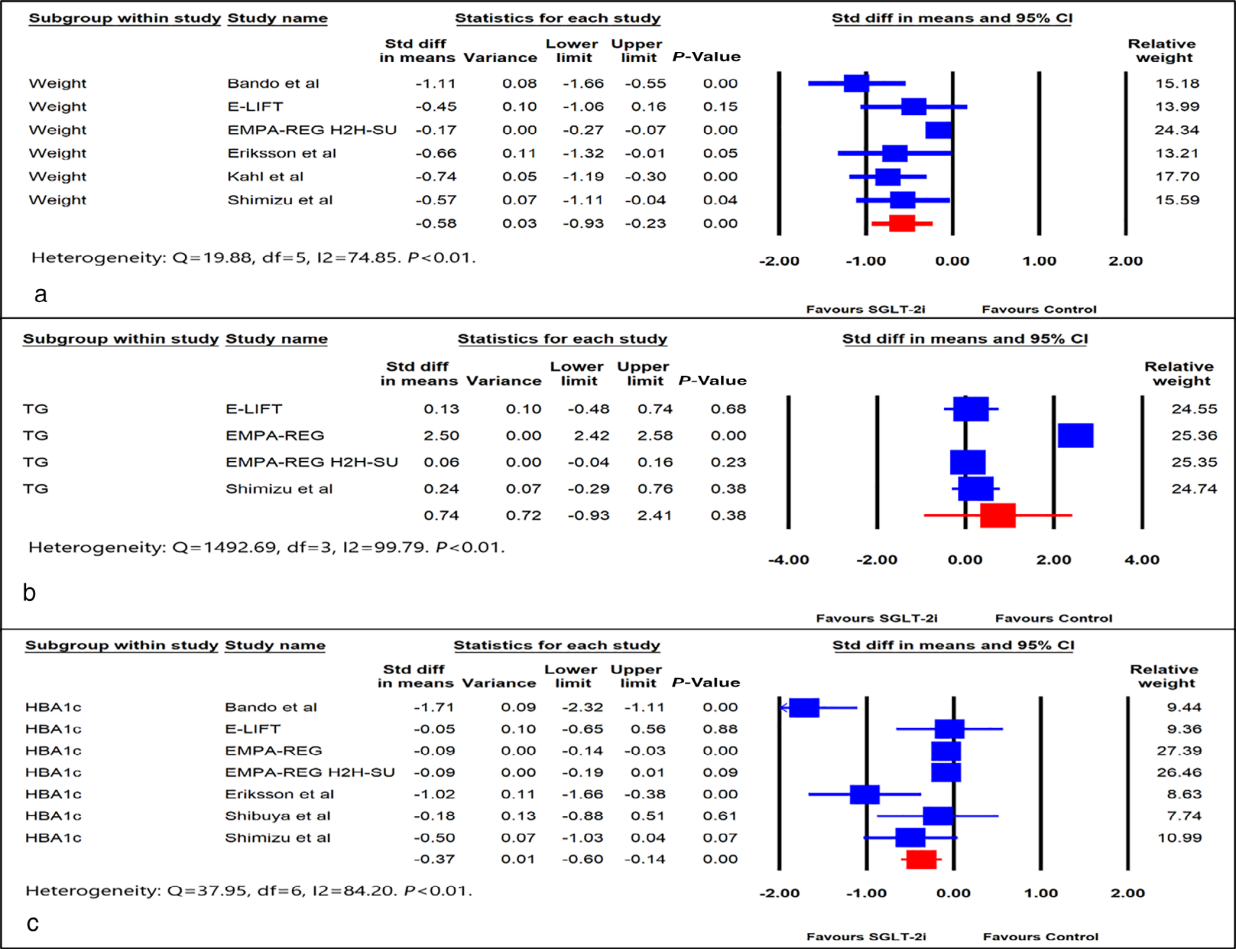
Forest plot comparing effect of SGLT‐2i *versus* control on: (a) weight, (b) triglyceride (TG), and (c) HBA1c. CI, confidence interval; Std, standard.

The citations included in the meta‐analysis reported changes in weight and BMI interchangeably. A couple of studies compared weight change related to the individual arms (SGLT‐2i and control) and not between them. As weight change was reported in significantly more trials than BMI, weight was included as the parameter of interest. The standardized difference in means of weight from baseline was in favor of the alternate hypothesis (SDM, −0.58, 95% CI, −0.93 to −0.23, *P* < 0.01) (Fig. 4a). The impact of SGLT‐2i on the decrease of TG was in favor of the null hypothesis (SDM, 0.74, 95% CI, −0.93 to 2.41, *P* = 0.38) (Fig. 4b). SGLT‐2i resulted in a significant reduction in HBA1c from baseline compared to the control arm (SDM, −0.37, 95% CI, −0.60 to −0.14, *P* < 0.01) (Fig. 4c). The mean difference in weight and HBA1c was −2.46 kg and −0.35%, respectively.

### *Sensitivity and subgroup analysis*

Significant heterogeneity was observed in some of the outcome measures (ALT, AST, HBA1c, weight, and TG). As a result, a sensitivity analysis was carried out on them by removing one study at a time and observing the reduction or disappearance of heterogeneity. Once the newly assessed heterogeneity was within an acceptable range, the outcome was reanalyzed to assess whether the result achieved previously could be replicated.

A pooled analysis of ALT had significant heterogeneity (I^2^ = 74.96). Three studies (*E*‐LIFT, Sibuya *et al*., and Bando *et al*.) contributed significantly to the heterogeneity. *Re*analyzing the pooled ALT data by removing these three studies significantly reduced heterogeneity (I^2^ = 48.85), without altering the positive impact of SGLT‐2i on ALT (SDM, −0.13, 95% CI, −0.17 to −0.07, *P* < 0.01) (Figure S2a). Only Bando *et al*. had significant heterogeneity as far as AST was concerned. Removing this study reduced the heterogeneity from I^2^ = 65.24 to I^2^ = 33.27, without altering the positive impact of SGLT‐2i on AST (SDM, −0.12, 95% CI, −0.17 to −0.07, *P* < 0.01) (Figure S2b).

The primary data with HbA1c dominated in the alternate hypothesis at the expense of significant heterogeneity (I^2^ = 84.2, *P* < 0.001), contributed by two studies (Bando *et al*. and Eriksson *et al*.). Removing the two studies resulted in the complete disappearance of heterogeneity (I^2^ = 0.000, *P* = 0.67) while retaining the original inference (SDM, −0.09, 95% CI, −0.14 to −0.04, *P* < 0.01) (Figure S2c).

With regard to weight, the EMPA‐REG 2H2‐SU study contributed the most to heterogeneity (I^2^ = 74.85, *P* = 0.001). Removing this study resulted in the disappearance of heterogeneity (I^2^ = 0.000, *P* = 0.56) while retaining the alternate hypothesis (SDM, −0.72, 95% CI, −0.96 to −0.48, *P* < 0.01) (Figure S2d). As far as TG was concerned, the major contribution to heterogeneity (I^2^ = 99.79, *P* = 0.001) came from the EMPA‐REG 2H2‐SU study. Removal of this study resulted in the complete disappearance of heterogeneity (I^2^ = 0.000, *P* = 0.79) while retaining the original outcome of accepting the null hypothesis (SDM, −0.07, 95% CI, −0.03 to 0.17, *P* = 0.16) (Figure S2e).

Because the SMD does not represent the effect size in the original units, we conducted a subgroup analysis of the important end‐points using the raw mean difference as effect size. This was not possible when including all the studies utilized in the SMD analysis because of different reporting patterns in some of the citations. The subgroup analysis was in line with the original data, with a significant reduction in ALT (−4.81 U/L, 95% CI, –7.28 to −2.34, *P* < 0.01) and AST (−2.00 U/L, 95% CI, –3.20 to −0.80, *P* < 0.01) (Figure S3a,b). There was significant improvement in weight (−1.57 kg, 95% CI, –2.03 to −1.11, *P* < 0.01) and HBA1c (−0.45%, 95% CI, –0.78 to −0.12, *P* = 0.01), endorsing the anticipated metabolic benefits (Figure S3c,e). The impact on TG was neutral (7.01 mg/dL, 95% CI, −2.39 to 16.41, *P* = 0.14) (Figure S3d).

Funnel plots were used to assess the extent of study bias, as well as the presence of outliers. (Figure S4).

## Discussion

### *Background knowledge*

NAFLD results from hepatic steatosis in the absence of heavy alcohol consumption and is one of the most common causes of chronic liver disease (CLD).^27^ It is strongly associated with metabolic syndrome—obesity, dyslipidemia, hypertension, insulin resistance, and T2D.^28^, ^29^ However, not all patients with metabolic risk factors will experience progression of liver disease, with prognostic markers from histological studies signaling that the degree of inflammation is the strongest and is an independent predictor for progression of liver fibrosis.^30^ Hence, therapies that can reduce hepatic inflammation, and thus hepatic fibrosis, would be strategic to control NAFLD, particularly in T2D.

Use of SGLT‐2i inhibitors has been shown to reduce weight and fat accumulation in animal models.^12^, ^13^ In humans, studies have confirmed that SGLT‐2i inhibitors can improve serum ALT, particularly in patients with high ALT levels.^31^ A recent meta‐analysis of six trials with 309 patients found that SGLT‐2i inhibitors can decrease ALT and liver fat content in patients with NAFLD independent of the hypoglycemic effect of SGLT‐2i inhibitors.^24^ This meta‐analysis was, however, restricted by an extremely small sample size and an inexplicable nonimprovement of glycemia despite the use of a powerful oral antidiabetic, although it is clearly documented that retardation in the progress of NAFLD in T2D is strongly associated with weight loss and reduction of HbA1C.^32^

### *Additional information from this meta‐analysis*

Our meta‐analysis was conducted on a large pooled population of 11 369 patients taken from 9 studies, with 7281 patients on SGLT‐2i inhibitors and a follow‐up period ranging from 12 to 28 weeks. There was a significant improvement in ALT (SDM, −0.21, 95% CI, −0.32 to −0.10, *P* < 0.01) and AST (SDM, −0.15, SE, 95% CI, −0.24 to −0.07, *P* < 0.01) levels, suggesting a decrease in hepatic inflammation. Improvement in liver fat and VFM, as measured by PDFF (SDM, −0.98, 95% CI, −1.53 to −0.44, *P* < 0.01) and VFM (SDM, −0.51, SE, 95% CI, −0.83 to −0.20, *P* < 0.01) showed statistical significance. This meta‐analysis also confirmed a significant benefit of weight loss (SDM, −0.58, 95% CI, −0.93 to −0.23, *P* < 0.01) and improvement of HbA1c (SDM, −0.37, 95% CI, −0.60 to −0.14, *P* < 0.01) in T2D patients with NAFLD on SGLT‐2i. No statistical significance was seen in the reduction of TG levels in these patients.

### *A comparative literature review*

An earlier study on diabetic rats revealed a beneficial effect of canagliflozin, an SGLT‐2i, in NAFLD by reducing inflammatory cytokines and oxidative stress in the liver.^33^ Moreover, in a study on nine diabetic patients with NAFLD, liver biopsy studies showed an improvement in steatosis and inflammation with canagliflozin, possibly driven by an improvement in glycemic parameters.^34^ The improvement in transaminase levels with SGLT‐2i, as clearly exhibited in this meta‐analysis, is strongly suggestive of a reduction in inflammation and oxidative stress, in keeping with the animal studies and histopathological data. This is likely due to the improvement in PDFF and VFM signaling reduction of hepatic fat accumulation, causing an increase in fatty acid oxidation leading to a reduction in hepatic inflammation. In contrast to the previous meta‐analysis, there is a statistically significant reduction of HbA1c along with body weight reduction, which are a hallmark of treatment with SGLT‐2i and are also potential mediators for the improvement in NAFLD as SGLT‐2i may augment the action of glucagon‐like peptide (GLP1).^35^ This mechanism may be important when considering any potential benefit to patients with NAFLD and T2D.

Hence, this is the first meta‐analysis confirming that SGLT‐2i inhibitors have a significant role in the treatment of T2D patients with NAFLD, probably driven by an improvement in inflammation in the liver by reducing liver fat accumulation and improving transaminase levels, which may retard the progression of liver disease. This improvement is likely to be a direct effect of the improvement in weight loss and glycemia. SGLT‐2i inhibitors may have additional antioxidant properties, which may decrease liver fat accumulation and inflammation, preventing or even improving liver fibrosis in NAFLD patients.^36^ Thus, in addition to lifestyle modification, we believe that SGLT‐2i are likely to play a major role in improving liver inflammation and liver fibrosis in the long term and may well be the cornerstone of the treatment of NAFLD with T2D.

### *Limitations and strengths*

This meta‐analysis has certain limitations. First, data were analyzed from the published effect size and not from individual‐level pooled data. This could have resulted in the loss of valuable patient‐related outcome information. Second, the outcomes of interest were reported in different formats. Some of the studies reported mean differences between SGLT‐2i and the control arm with its associated statistical significance and CI, while others reported the mean changes in the individual arms only. A few studies did not mention any level of significance. In view of such heterogeneous reporting, an SMD was calculated for this meta‐analysis instead of the raw mean difference, which is easier to correlate. Most importantly, the absence of biopsy‐proven improvements in hepatic architecture and function was a major limitation as liver biopsy remains the gold‐standard diagnostic test, yet it is performed relatively rarely due to associated risks.

The major strengths of this meta‐analysis were the large number of patients included from RCTs and uniform reporting of all outcomes of interest. Studies that included agents like pioglitazone or GLP1‐RA in the control arm, which are known to have a positive impact on hepatic outcomes, were excluded to ensure a more accurate measure of the effects of SGLT‐2i. Moreover, all RCTs published till date were included in this meta‐analysis.

In conclusion, the improvement of hepatic inflammation and fat accumulation, possibly driven by an improvement in body weight and glycemia, makes SGLT‐2i a potent and novel option for the treatment of patients with T2D and NAFLD. However, as there is no long‐term data on patients with NAFLD and T2D using SGLT‐2i inhibitors, long‐term RCTs with liver biopsies or at least liver elastography before and after treatment will be necessary to confirm the effects of SGLT‐2i inhibitors in NAFLD patients with T2D.

## Supporting information


**Figure S1.** Assessment of quality of the citations selected for meta‐analysis using the Cochrane risk‐of‐bias algorithm.

**Figure S2.** Sensitivity analysis with forest plot comparing effect (SMD) of SGLT‐2i *versus* control on: (a) ALT (alanine aminotransferase), (b) AST (aspartate aminotransferase), (c) HBA1c, (d) weight, and (e) TG (triglyceride), CI, confidence interval; Std, standard.

**Figure S3.** Subgroup analysis with forest plot comparing effect (mean difference) of SGLT‐2i *versus* control on: (a) ALT (alanine aminotransferase), (b) AST (aspartate aminotransferase), (c) weight, (d) TG (triglyceride), and (e) HBA1c.

**Figure S4.** Funnel plot assessing publication bias. (a) ALT (Alanine aminotransferase), (b) AST (Aspartate aminotransferase), (c) liver fat, and (d) weight.
