# Supplementary figures and images for: Meta‐analysis of the effects of sodium glucose cotransporter 2 inhibitors in non‐alcoholic fatty liver disease patients with type 2 diabetes
Source: JGH Open. 2020 Dec 7;5(2):219–27. doi: 10.1002/jgh3.12473 (PMC7857274; doi:10.1002/jgh3.12473)

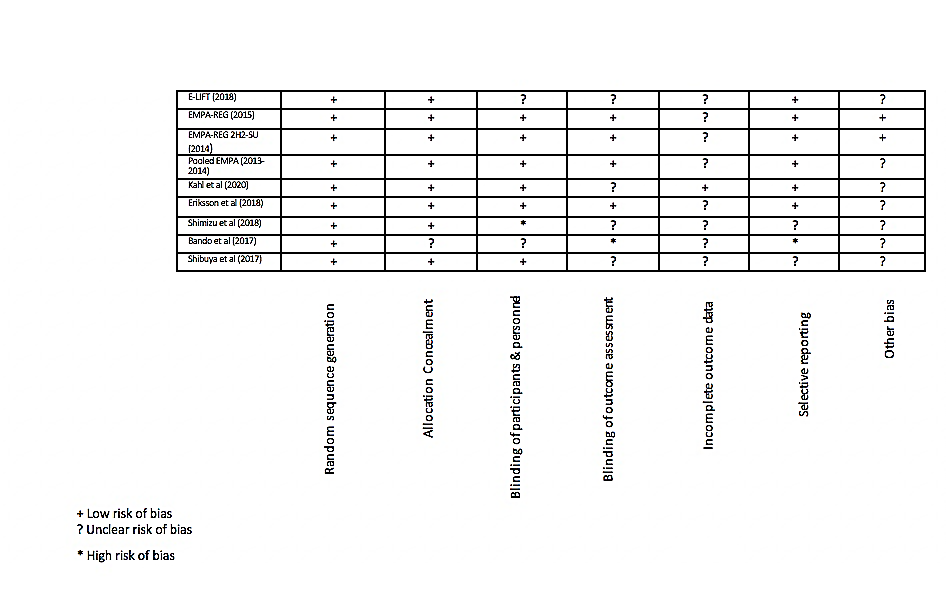

Supplement: Supplementary file 1 — Figure S1. Assessment of quality of the citations selected for meta‐analysis using the Cochrane risk‐of‐bias algorithm. [file JGH3-5-219-s001.tiff]

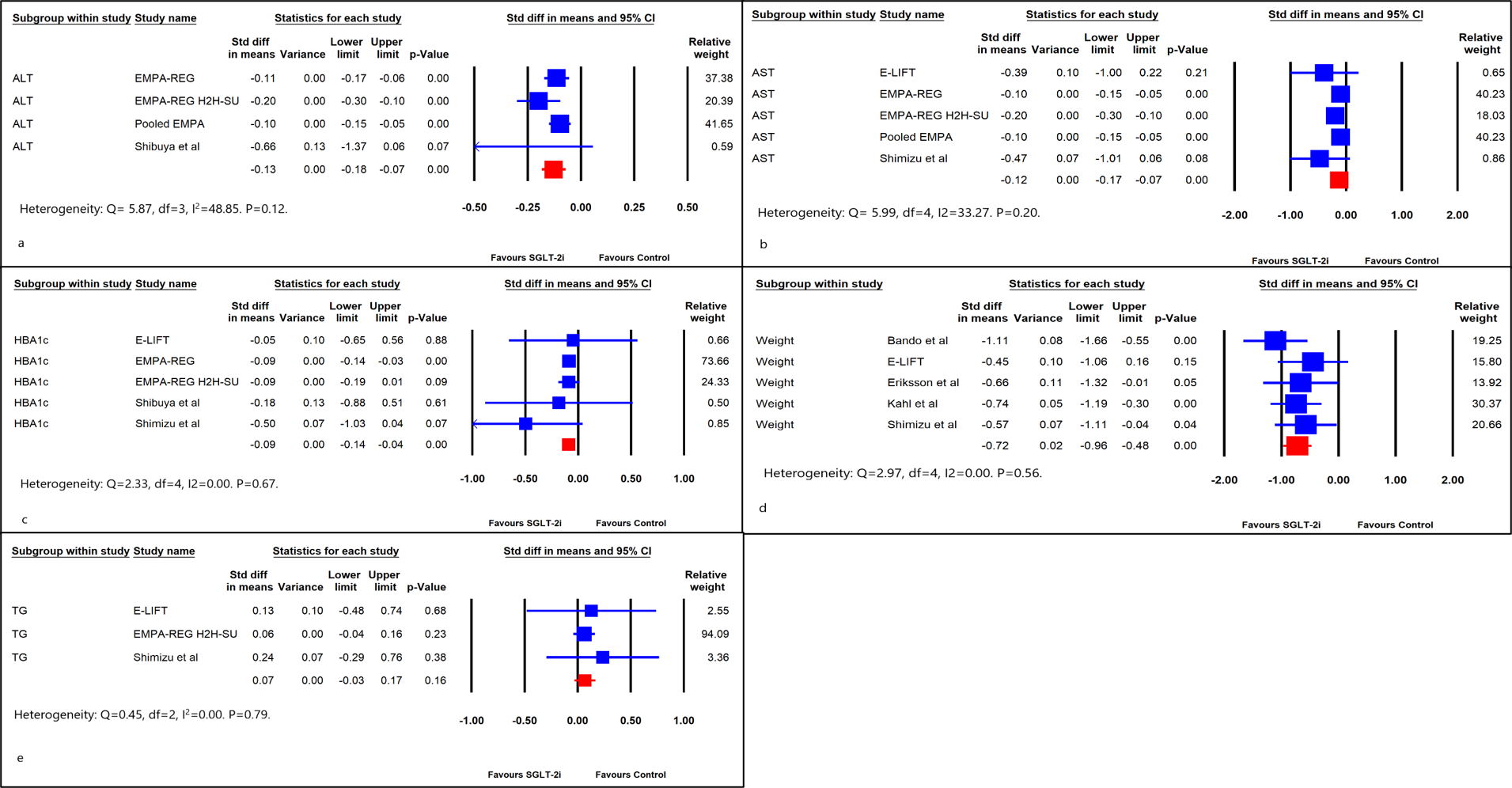

Supplement: Supplementary file 2 — Figure S2. Sensitivity analysis with forest plot comparing effect (SMD) of SGLT‐2i versus control on: (a) ALT (alanine aminotransferase), (b) AST (aspartate aminotransferase), (c) HBA1c, (d) weight, and (e) TG (triglyceride), CI, confidence interval; Std, standard. [file JGH3-5-219-s002.tif]

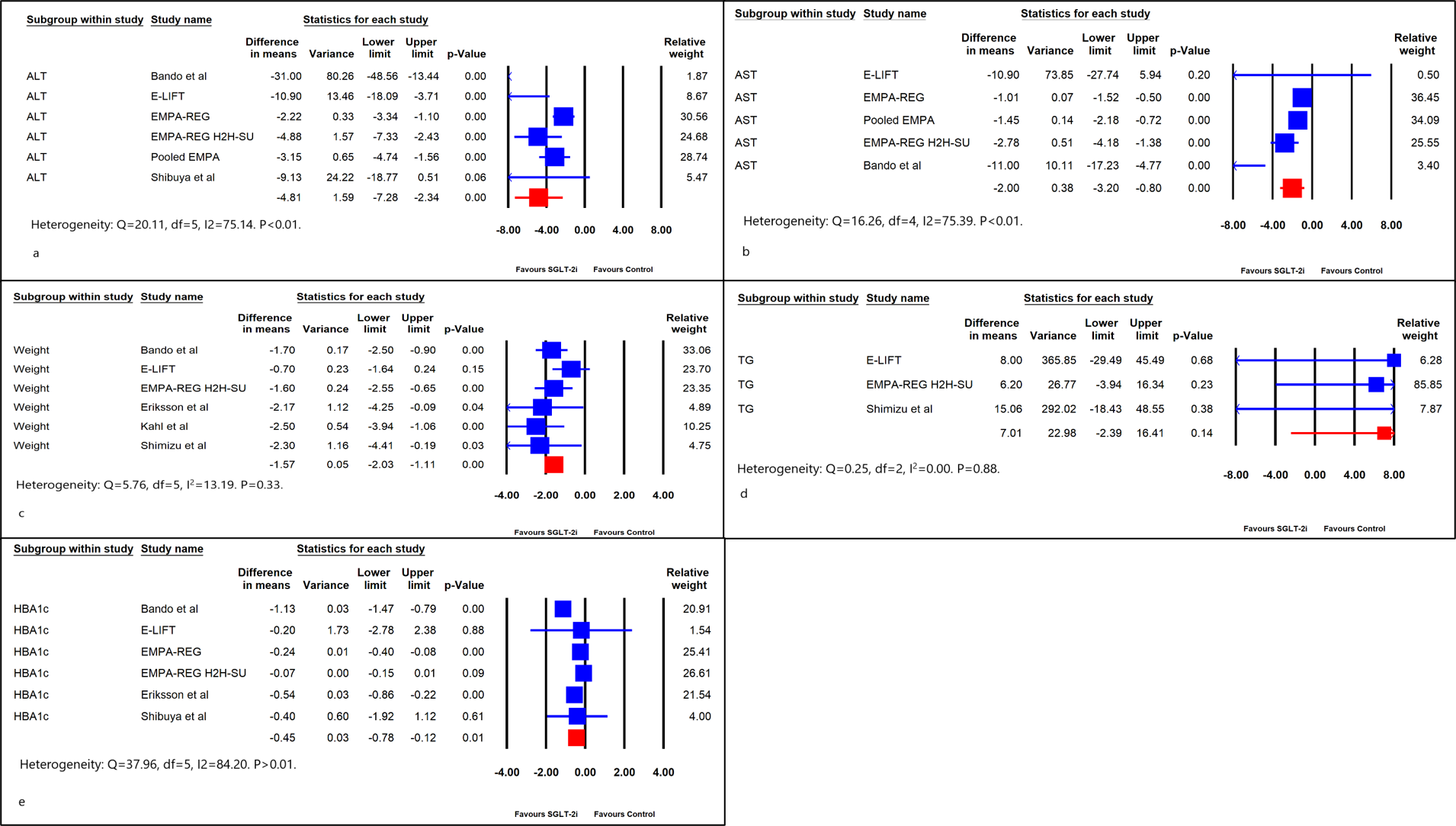

Supplement: Supplementary file 3 — Figure S3. Subgroup analysis with forest plot comparing effect (mean difference) of SGLT‐2i versus control on: (a) ALT (alanine aminotransferase), (b) AST (aspartate aminotransferase), (c) weight, (d) TG (triglyceride), and (e) HBA1c. [file JGH3-5-219-s003.tif]

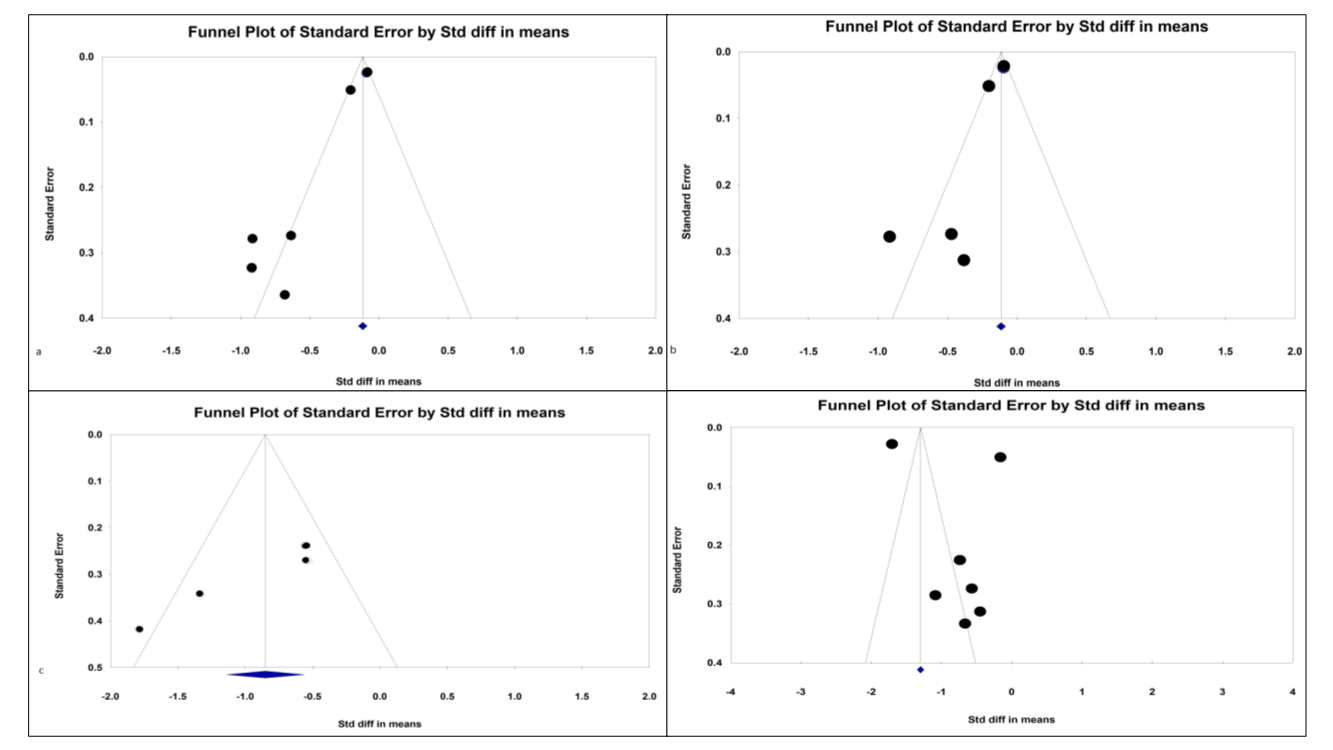

Supplement: Supplementary file 4 — Figure S4. Funnel plot assessing publication bias. (a) ALT (Alanine aminotransferase), (b) AST (Aspartate aminotransferase), (c) liver fat, and (d) weight. [file JGH3-5-219-s004.tiff]
